# Continuous mode adaptation for cable-driven rehabilitation robot using reinforcement learning

**DOI:** 10.3389/fnbot.2022.1068706

**Published:** 2022-12-22

**Authors:** Renyu Yang, Jianlin Zheng, Rong Song

**Affiliations:** ^1^Key Laboratory of Sensing Technology and Biomedical Instrument of Guangdong Province, School of Biomedical Engineering, Sun Yat-sen University, Guangzhou, China; ^2^School of Biomedical Engineering, Shenzhen Campus of Sun Yat-sen University, Shenzhen, China

**Keywords:** admittance control, cable-driven rehabilitation robot, human-robot cooperation, human-robot interaction, optimal control, robot compliance

## Abstract

Continuous mode adaptation is very important and useful to satisfy the different user rehabilitation needs and improve human–robot interaction (HRI) performance for rehabilitation robots. Hence, we propose a reinforcement-learning-based optimal admittance control (RLOAC) strategy for a cable-driven rehabilitation robot (CDRR), which can realize continuous mode adaptation between passive and active working mode. To obviate the requirement of the knowledge of human and robot dynamics model, a reinforcement learning algorithm was employed to obtain the optimal admittance parameters by minimizing a cost function composed of trajectory error and human voluntary force. Secondly, the contribution weights of the cost function were modulated according to the human voluntary force, which enabled the CDRR to achieve continuous mode adaptation between passive and active working mode. Finally, simulation and experiments were conducted with 10 subjects to investigate the feasibility and effectiveness of the RLOAC strategy. The experimental results indicated that the desired performances could be obtained; further, the tracking error and energy per unit distance of the RLOAC strategy were notably lower than those of the traditional admittance control method. The RLOAC strategy is effective in improving the tracking accuracy and robot compliance. Based on its performance, we believe that the proposed RLOAC strategy has potential for use in rehabilitation robots.

## 1 Introduction

Stroke is one of the leading causes of neurological and functional disability. In China, two million people suffer from stroke each year. Rehabilitation robots have attracted tremendous interest among researchers globally, as they can provide high-intensity, repetitive, and interactive rehabilitation training for post-stroke patients and overcome the labor-intensiveness of traditional manual rehabilitation training (Kwakkel et al., 2008). Rehabilitation robots, including various exoskeleton-type rehabilitation robots, such as ARMin (Nef et al., 2007), RUPERT (Huang et al., 2016), and UL-EXO7 (Kim et al., 2013) mimic the role of therapists to provide assistive forces to each joint of the human arm in rehabilitation training. However, these exoskeletons with hulking rigid links and motors attached to the human arm significantly increase the movement inertia, resulting in change in human arm dynamics, which will reduce the transparency of human–robot interaction (HRI) (Mao et al., 2015). To reduce the moving mass of the robot, a novel rehabilitation robot called cable-driven rehabilitation robot (CDRR), wherein the end-effectors are driven by cables instead of hulking rigid links, was developed, which improved the HRI performance owing to its excellent characteristics of low inertia, compliant structure, safety, and transparency (Jin et al., 2018). Mao et al. (2015) developed a cable driven exoskeleton (CAREX) for upper arm rehabilitation, which uses multi-stage cable-driven parallel mechanism to reduce the movement inertia, and the feasibility was verified in patients. Alamdari and Krovi (2015) designed a home-based cable-driven parallel platform robot driven by five cables for upper-limb neuro-rehabilitation in three-dimensional space. Cui et al. (2017) designed a 7-degrees of freedom (DOFs) cable-driven arm exoskeleton can easily assist the upper limbs to realize complex training tasks, involving rotation, translation, and their combination. Chen et al. (2019) designed a cable-driven parallel waist rehabilitation robot and a two-level control algorithm was proposed to assist patients with waist injuries to perform rehabilitation training.

The control strategies applied in rehabilitation robots play a critical role in the rehabilitation effectiveness (Guanziroli et al., 2019). According to the different recovery stages of post-stroke patients, the control strategies mainly include passive and active control (Proietti et al., 2016). Passive control is generally used to drive the patient repetitively move along predefined trajectories to improve the movement ability and reduce muscle atrophy, which is commonly adopted in the early recovery stages for patients with severe impairment (Jamwal et al., 2014). In active control, the rehabilitation robot assists the patient by complying with human motion intentions; it is mainly applied to patients with mild impairment. Koenig and Riener (2016) pointed out that passive control ignores the patient’s voluntary engagement, which is one of the essential factors to facilitate neuroplasticity and motor function recovery of post-stroke patients (Warraich and Kleim, 2010), so its effect of stimulating neuroplasticity is limited. Performance-adaptive control strategies for patients with different levels of motor disabilities are necessary to meet user rehabilitation needs and recovery stages (Sainburg and Mutha, 2016). Meuleman et al. (2016) developed a variable admittance control for LOPES II, which can implement both active control to passive control. Wolbrecht et al. (2008) proposed an assist-as-needed (AAN) control strategy to allow robots to provide only essential assistance according to the patient’s movement performance.

Obtaining suitable impedance/admittance parameters for the control strategy is essential to improve HRI performance for rehabilitation robots. The bio-inspired method assuming fixed impedance such as the musculoskeletal model (Pfeifer et al., 2012) or measurements of biological joint impedance (Erden and Billard, 2015) was used to estimate the impedance parameters through offline identification. The linear quadratic regulator (LQR) was adopted to obtain the desired admittance parameters through a cost function (Matinfar and Hashtrudi-Zaad, 2016). These methods would be good candidates when accurate models are available and their parameters can be well estimated. It is not practically applicable in rehabilitation training scenarios, because it is difficult to build the human dynamics model due to its features of nonlinearity, complexity, and variability (Driggs-Campbell et al., 2018). In addition, modeling and measurement errors are inevitable. To deal with this problem, the reinforcement learning (RL) algorithm was used to solve the given LQR problem, minimizing a cost function for optimizing the overall human–robot system performance (Modares et al., 2016; Li et al., 2017). RL algorithms have shown unprecedented successes in solving optimal control policy problems such as deep RL, including several policy search methods and deep Q-network (DQN) (Mnih et al., 2015; Silver et al., 2016). Doya (2000) used the knowledge of the system models to learn the optimal control policy and extend to continuous-time systems. To handle unknown dynamics, adaptive dynamic programming (ADP) with special a critic–actor structure has been extensively studied (Vrabie et al., 2009; Jiang and Jiang, 2012; Modares et al., 2015), which has become a promising tool for learning impedance/admittance parameters for the human–robot system. The ADP-based RL (ADPRL) approach was employed to automatically tune 12 impedance parameters and configure a robotic knee with human-in-the-loop (Wen et al., 2019; Gao et al., 2021). To achieve a compliant physical robot–environment interaction, Peng et al. (2022) used the ADPRL approach to obtain the desired admittance parameters based on the cost function composed of interaction force and trajectory tracking without the knowledge of the environmental dynamics. However, a fixed contribution weight of the cost function was adopted in previous studies, which cannot achieve continuous mode adaptation between the passive and active working mode.

Continuous mode adaptation is very important and useful to satisfy the different user rehabilitation needs and improves human–robot interaction (HRI) performance for rehabilitation robots. In this study, we present a novel reinforcement-learning-based optimal admittance control (RLOAC) strategy, which can achieve on-the-fly transitions between the passive and active working mode according to the human voluntary force. Firstly, we employed an RL algorithm to calculate the optimal admittance parameters for adapting to the different needs of patients without prior knowledge of the human dynamics model and formulated a new control strategy, which applied the optimal admittance parameters real time by minimizing the cost function to realize the desired HRI performance. Secondly, to promote patients’ voluntary engagement, the contribution weights of the cost function were adjusted according to the human voluntary force.

## 2 Control strategy design

### 2.1 RLOAC framework

**FIGURE 1:**
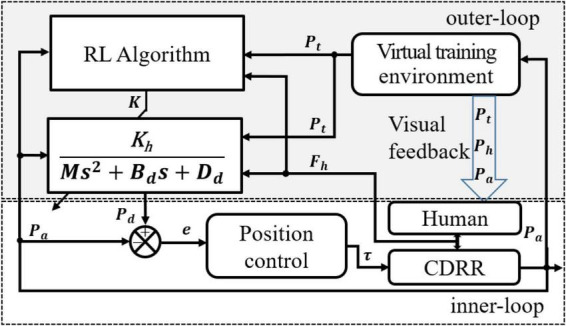
The proposed control framework.

The RLOAC framework consists of two control loops—inner loop and outer loop—as illustrated in Figure 1. The inner-loop is intended for position control, which compensates for the robot nonlinear dynamics and guarantees trajectory tracking accuracy and stability. This module was implemented and reported in our previous work (Yang et al., 2022). The outer loop includes three modules: (1) a virtual training environment module provides visual feedback of the trajectory tracking and obstacle avoidance (TTOA) movement task to the subject and outputs the predefined trajectory *P_t*, detailed in Section “4.2 Adaptation to human dynamics”; (2) an optimal admittance control method is employed to yield the desired trajectory *P_d* to obtain the optimal HRI performance according to the human voluntary force *F_h*; and (3) an RL algorithm is designed to calculate the optimal parameters ***K*** online, considering that the human and robot dynamics parameters are difficult to identify in practice. The details of the outer-loop designs are presented below.

### 2.2 Optimal admittance control

The predefined trajectory *P*_*t*_ ∈ ℝ^*n*^ set by the therapist, which is outputted directly to the CDRR, can be expressed in the form of a state equation in the Cartesian space.


(1) $${\dot{\bar{\text{P}}}}_{t}=\text{A}\,{\bar{\text{P}}}_{t}+\text{B}\,{\ddot{\bar{\text{P}}}}_{t},$$

(2) $$\text{A}=\left[\begin{array}{cc} 0 & I_{n}\\ 0 & 0 \end{array}\right],\text{B}=\left[\begin{array}{c} 0\\ I_{n} \end{array}\right],{\bar{P}}_{t}={\left[P_{t}\,{\dot{P}}_{t}\right]}^{T}$$

The relationship between the human voluntary force *F*_*h*_ and the movement of the end-effector *P*_*d*_ can be described by the following admittance model (Modares et al., 2016; Li et al., 2017):


(3) $$M_{d}\,{\ddot{P}}_{d}+B_{d}\,{\dot{P}}_{d}+D_{d}\,P_{d}=K_{h}\,F_{h}+l\,\left(P_{t}\right)$$

where *M*_*d*_,*B*_*d*_,*D*_*d*_, and *K*_*h*_ are the inertia, damping, stiffness, and proportional gain matrices of the human voluntary force, respectively; *l*(*P*) is an auxiliary input term, which will be designed later. By defining the augmented state **${\bar{P}}_{d}={\left[P_{d},{\dot{P}}_{d}\right]}^{T}$**, (3) is expressed in the form of a state equation as


(4) $${\dot{\bar{P}}}_{d}=A\,{\bar{P}}_{d}+B\,u,$$

where A and B are defined as in (2), and *u*ϵ^ℝ*m*^. Combining (3) and (4) *u* is expressed as


(5) $$u=M_{d}^{-1}\,\left(-B_{d}\,{\dot{P}}_{d}-D_{d}\,P_{d}+K_{h}\,F_{h}+l\,\left(P_{t}\right)\right)$$

The trajectory deformations are defined as *e*_*d*_=*P*_*t*_−*P*_*d*_ and ${\bar{e}}_{d}={\left[e_{d},{\dot{e}}_{d}\right]}^{T}$. Combining (1) and (4), the trajectory deformation dynamics is expressed as


(6) $$\dot{{\bar{e}}_{d}}=A\,{\bar{e}}_{d}+B\,\left({\ddot{\bar{P}}}_{t}-u\right).$$

Similar to the approach in Suzuki and Furuta (2012), human dynamics is expressed as


(7) $$\dot{F_{h}}=-T^{-1}\,F_{h}+T^{-1}\,K_{d}\,\dot{e_{d}}+T^{-1}\,K_{p}\,e_{d},$$

where *K*_*p*_, *K*_*d*_, and *T* are proportional coefficient of the human brain controller, differential coefficient, and time constant of the neuromuscular system, respectively. Defining the state variate as $X={\left[e_{d},{\dot{e}}_{d}\,F_{h}\right]}^{T}$ and then combining (6) and (7), a state equation for the HRI system can be established as


(8) $$\dot{F_{h}}=-T^{-1}\,F_{h}+T^{-1}\,K_{d}\,\dot{e_{d}}+T^{-1}\,K_{p}\,e_{d},$$

(9) $$\bar{A}=\left[\begin{array}{ccc} 0 & I_{n} & 0\\ 0 & 0 & 0\\ T^{-1}\,K_{p} & T^{-1}\,K_{d} & -T^{-1} \end{array}\right],\bar{B}=\left[0\,I_{n}\,0\right],$$

$$u=M_{d}^{-1}\,\left(-B_{d}\,{\dot{P}}_{d}-D_{d}\,P_{d}+K_{h}\,F_{h}+l\,\left(P_{t}\right)\right)$$

$$=M_{d}^{-1}\,\left(B_{d}\,{\dot{e}}_{d}+D_{d}\,e_{d}+K_{h}\,F_{h}\right)$$

$$+M_{d}^{-1}\,\left(l\,\left(P_{t}\right)-M_{d}\,{\ddot{P}}_{t}-B_{d}\,{\dot{P}}_{t}-D_{d}\,P_{t}\right)$$

(10) $$\equiv u_{e}+u_{d}$$

The control input *u* can be divided into two elements, feedback control input *u*_*e*_ and feedforward control input *u*_*d*_(Modares et al., 2016; Li et al., 2017). We designed the auxiliary input term *l*(*P*_*t*_) in (10) as


(11) $$l\,\left(P_{t}\right)=M_{d}\,{\ddot{P}}_{t}+B_{d}\,{\dot{P}}_{t}+D_{d}\,P_{t}.$$

Then, (10) can be rewritten as


(12) $$\dot{X}=\bar{A}\,X+\bar{B}\,u_{e}.$$

**FIGURE 2:**
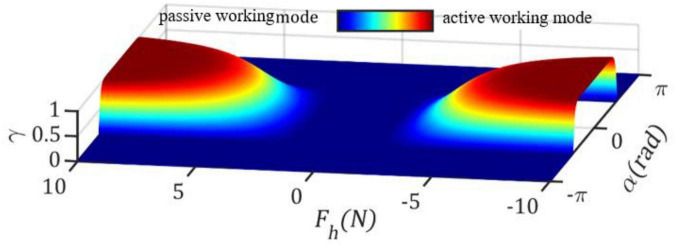
Smooth transition of weight factor γ(*F*_*h*_,α) between 0 and 1. γ(*F*_*h*_,α) = 0 and γ(*F*_*h*_,α) = 0 correspond to passive and active working mode, respectively.

The feedback control input can be rewritten as


(13) $$u_{e}=K\,X,K=M_{d}^{-1}\,\left[\begin{array}{ccc} B_{d} & D_{d} & K_{h} \end{array}\right],$$

where *K* ∈ ℝ*^3n×3n^* is the control gain, which contains the admittance parameters. To minimize *e*_*d*_, $\dot{e_{d}}$, *u*_*e*_, and *F*_*h*_, a cost function is designed as follows:


(14) J=∫0∞(edT⁢Q1⁢ed+ed.T⁢Q2⁢ed.+ueT⁢R1⁢ue+FhT⁢R2⁢Fh)⁢dt,

where *Q*_1_,*Q*_2_,*R*_1_,*R*_2_ ∈ ℝ*^n = n^* are the weighting factors of $e_{d},\dot{e_{d}},u_{e},$ and *F*_*h*_, which allow a trade-off between the tracking error and human voluntary force. *Q* and *R*_2_are defined as follows:


(15) $$Q=\left[\begin{array}{ccc} Q_{1} & 0 & 0\\ 0 & Q_{2} & 0\\ 0 & 0 & R_{2} \end{array}\right],R_{2}=d\,i\,a\,g\,\left(r_{2},\cdots ,r_{2}\right),$$

where *r*_2_ is the diagonal element of *R*_2_. *R*_1_ and *R*_2_ determine the relative contributions of shared control between human and robot, respectively, to the cost *J*. Robotic systems are capable of adaptation of their autonomy level through dynamical adjustment of *R*_2_. A smaller *R*_2_ indicates a higher propensity for robots to lead the shared control task, vice versa. Since the motion capability and intention of the subject can be estimated by her/his voluntary force, *R*_2_ should be adjusted according to human voluntary force to improve HRI performance in terms of robot compliance. A larger human voluntary force indicates a stronger capability and motion intentions to deviate the trajectory from the predefined trajectory. In this case, humans should be assigned the dominant role whereas the robots show greater compliance with the human voluntary actions, which can be achieved by increasing *R*_2_. The reverse is true for a smaller human voluntary force. Thus, by modulating *R*_2_ according to the human voluntary force, robots can realize continuous mode adaptation between passive and active working mode. The weighting element *r*_2_ can be adjusted as follows:


(16) $$r_{2}=\left\{ \begin{array}{c} r_{m\,i\,n}+\gamma \,\left(F_{h},\alpha \right)\,\left(r_{m\,a\,x}-r_{m\,i\,n}\right),i\,f\,{||F_{h}||}_{2}>F_{c},\alpha \in \left(-\frac{\pi }{2},\frac{\pi }{2}\right)\\ r_{m\,i\,n},o\,t\,h\,e\,r\,w\,i\,s\,e \end{array}\right.$$

where *r*_*min*_, *r*_*max*_ are the minimum and maximum values of *r*_*2*_. *F_c* is the threshold value of the human voluntary force. ||⋅|_|2_ denotes the 2-norm of a vector. ||*F*_*h*_||_2_≤*F*_*c*_ implies that *F_h* contains only sensor noises or involuntary force, which means the user cannot exert a voluntary force and therefore the CDRR should operate in the passive working mode. α is the magnitude of the directional difference between *F_h* and the optimal control input ue*. The condition $\alpha \in \left(-\frac{\pi }{2},\frac{\pi }{2}\right)$ indicates that the direction of *F_h* agrees with that of ue*. The conditions $||F_{h}||>F_{c}\,\mathrm{and}\,\alpha \in \left(-\frac{\pi }{2},\frac{\pi }{2}\right)$ indicate that the user has some capability to correctly perform the cooperative control tasks; hence, the CDRR should operate in the active working mode. γ(*F*_*h*_,α) ∈ [0,1] is a weight factor, which is used to transit *r*_*2*_ smoothly between *r*_*min*_ and *r*_*max*_ and is defined as


(17) $$\gamma \,\left(F_{h},\alpha \right)=t\,a\,n\,h\,\left(\mu \cdot m\,a\,x\,{\left\{0,{||F_{h}||}_{2}-{||F_{c}||}_{2}\right\}}^{2}\cdot m\,a\,x\,\left(0,c\,o\,s\,\alpha \right)\right),$$

where μ is a scale factor, which determines the ramping rate of γ. The weight factor γ(*F*_*h*_,α) for μ=0.5,*F*_*c*_=1.5*N* is illustrated in Figure 2.

Based on the optimal theorem (Kwakernaak and Sivan, 1972), the optimal admittance parameters can be obtained using the LQR algorithm with the exact model parameters of the human control and robot system dynamics. The optimal parameters that minimize the cost function (14) are given by


(18) $$K^{{}^{*}}=-R_{1}^{-1}\,{\bar{B}}^{T}\,P^{{}^{*}},u_{e}^{{}^{*}}=K^{{}^{*}}\,X,$$

where P* is the solution to the following algebraic Riccati equation (ARE):


(19) $${\bar{A}}^{T}\,P+P\,\bar{A}-P\,\bar{B}\,R_{1}^{-1}\,{\bar{B}}^{T}\,P+Q=0.$$

Thus, the optimal admittance parameters and proportional gain of the human voluntary force (*M*_*d*_, *B*_*d*_,*D*_*d*_, and *K_h*) are determined.

### 2.3 RL algorithm

The disadvantage of solving the ARE (19) by using the LQR algorithm is that it requires the exact parameters of the human–robot system dynamics, which is difficult to know in practice. Several RL algorithms have been designed to overcome this limitation (Vrabie et al., 2009; Jiang and Jiang, 2012; Modares and Lewis, 2014). In this study, the RL algorithm (Jiang and Jiang, 2012) was employed for online calculation of the optimal admittance parameters for adapting to the needs of different patients under the human–robot system dynamics parameters completely unknown. Based on Theorem 2 in Jiang and Jiang (2012), the numerical approximation form of the Bellman equation for the aforementioned LQR problem of the system in (12) to solve the ARE (19) is given below.


(20) XT⁢(t+δ⁢t)⁢Pk⁢X⁢(t+δ⁢t)-XT⁢(t)⁢Pk⁢X⁢(t)=-∫tt+δ⁢tXT⁢(τ)⁢[Q+(Kk)T⁢R1⁢Kk]⁢X⁢(τ)⁢dτ+2⁢∫tt+δ⁢tueT⁢(τ)⁢R1⁢Kk+1⁢X⁢(τ)⁢dτ+2⁢∫tt+δ⁢t[Kk⁢X⁢(τ)]TR1⁢Kk+1⁢X⁢(τ)⁢d⁢τ

It is clear that (20) does not rely on the dynamic parameters $\bar{A}$ or $\bar{B}$ in (10). Then, the Kronecker product is used to express (20) as (Jiang and Jiang, 2012)


(21) $$X^{T}\,\left(t+\delta \,t\right)\,P^{k}\,X\,\left(t+\delta \,t\right)={\bar{X}}^{T}\,\left(t+\delta \,t\right)\,{\bar{P}}^{k},$$

(22) $$X^{T}\,\left(t\right)\,P^{k}\,X\,\left(t\right)={\bar{X}}^{T}\,\left(t\right)\,{\bar{P}}^{k},$$

$$X^{T}\,\left(\tau \right)\,\left[Q+{\left(K^{k}\right)}^{T}\,R_{1}\,K^{k}\right]\,X\,\left(\tau \right)$$

(23) $$=X^{T}\,\left(\tau \right)⊗X^{T}\,\left(\tau \right)\,v\,e\,c\,\left(Q+{\left(K^{k}\right)}^{T}\,R_{1}\,K^{k}\right),$$

ueT⁢(τ)⁢R1⁢Kk+1⁢X⁢(τ)

(24) $$=\left[u_{e}^{T}\,\left(\tau \right)⊗X^{T}\,\left(\tau \right)\right]\,\left(R_{1}⊗I_{n}\right)\,v\,e\,c\,\left(K^{k+1}\right),$$

$${\left[K^{k}\,X\,\left(\tau \right)\right]}^{T}\,R_{1}\,K^{k+1}\,X\,\left(\tau \right)$$

$$=\left[X^{T}\,\left(\tau \right)⊗X^{T}\,\left(\tau \right)\right]\,\left[I_{n}⊗{\left(K^{k+1}\right)}^{T}\,R_{1}\right]\,v\,e\,c\,\left(K^{k+1}\right),$$

Where


$$X=\left[X_{1}\,\cdots \,X_{n}\right],$$

$$\bar{X}=\left[X_{1}^{2},X_{1}\,X_{2},\cdots ,X_{1}\,X_{n},X_{2}^{2},X_{2}\,X_{3},\cdots ,X_{n-1}\,X_{n},X_{n}^{2}\right]$$

(26) $${\bar{P}}^{k}=\left[P_{11}^{k},2\,P_{12}^{k},\cdots ,2\,P_{1\,n}^{k},P_{22}^{k},2\,P_{23}^{k},\cdots ,2\,P_{n-1,n}^{k},P_{n\,n}^{k}\right]$$

Combining (21) and (22), the left-hand side of (20) can be written as


$$X^{T}\,\left(t+\delta \,t\right)\,P^{k}\,X\,\left(t+\delta \,t\right)-X^{T}\,\left(t\right)\,P^{k}\,X\,\left(t\right)$$

(27) $$=\left[{\bar{X}}^{T}\,\left(t+\delta \,t\right)-{\bar{X}}^{T}\,\left(t\right)\right]\,{\bar{P}}^{k}$$

By combining (21)–(25), (20) can be rewritten as


(28) $$\begin{array}{c} \left[{\bar{X}}^{T}\,\left(t+\delta \,t\right)-{\bar{X}}^{T}\,\left(t\right)\right]\,{\bar{P}}^{k}\\ =-v\,e\,c\,\left(Q+{\left(K^{k}\right)}^{T}\,R_{1}\,K^{k}\right)\,\int_{t}^{t+\delta \,t}X^{T}⊗X^{T}\,d\tau \\ +2\,\left(R_{1}⊗I_{n}\right)\,v\,e\,c\,\left(K^{k+1}\right)\,\int_{t}^{t+\delta \,t}u_{e}^{T}⊗X^{T}\,d\tau \\ +2\,\left[I_{n}⊗{\left(K^{k+1}\right)}^{T}\,R\right]\,v\,e\,c\,\left(K^{k+1}\right)\,\int_{t}^{t+\delta \,t}X^{T}⊗X^{T}\,d\tau \end{array}$$

We introduce the following definitions to reduce (27) into a simple form:


(29) $$\begin{array}{cc} \delta_{X\,X} & ={\bar{X}}^{T}\,\left(t+\delta \,t\right)-{\bar{X}}^{T}\,\left(t\right),\\ I_{X\,X} & =\int_{t}^{t+\delta \,t}X^{T}⊗X^{T}\,d\tau ,\\ I_{X\,u} & =\int_{t}^{t+\delta \,t}X^{T}⊗X^{T}\,d\tau ,\\ b^{k} & =-{\text{I}}_{X\,X}\,v\,e\,c\,\left(Q+{\left(K^{k}\right)}^{T}\,R_{1}\,K^{k}\right),\\ \Gamma^{k} & =\left[\delta_{X\,X},-2\,{\text{I}}_{X\,X}\,\left({\text{I}}_{n}⊗{\left(K^{k+1}\right)}^{T}\,R_{1}\right)-2\,{\text{I}}_{X\,u}\,\left(R_{1}⊗{\text{I}}_{n}\right)\right]. \end{array}$$

Then, (27) can be simplified as


(30) $$\begin{array}{c} \Gamma^{k}\,\left[\begin{array}{c} {\bar{P}}^{k}\\ v\,e\,c\,\left(K^{k+1}\right) \end{array}\right]=b^{k} \end{array}$$

Refer to study (Jiang and Jiang, 2012), a least-squares (LS) method is implemented online to obtain the optimal solution P*. First, set *u*_*e*_=*K*^0^ + φ as the initial input. *K*^0^ is the initial value of the control gain. φ is a probing noise. Then, the online data are collected and δ_*XX*_, *I*_*XX*_, and *I*_*Xu*_ are calculated until the following rank condition is satisfied:


(31) $$r\,a\,n\,k\,\left(\left[I_{X\,X},I_{X\,u}\right]\right)=\frac{3\,n\,\left(3\,n+1\right)}{2}+3\,m\,n$$

After the rank condition is satisfied, the LS solution is obtained as


(32) $$\left[\begin{array}{c} {\bar{P}}^{k}\\ v\,e\,c\,\left(K^{k+1}\right) \end{array}\right]={\left[{\left(\Gamma^{k}\right)}^{T}\,\Gamma^{k}\right]}^{-1}\,{\left(\Gamma^{k}\right)}^{T}\,b^{k}$$

Then, the policy is improved as *u*_*e*_=*K^k + 1^X* and the above procedure of LS is repeatedly implemented until ||*K^k + 1^*−^*Kk*^|| < ε. Finally, the optimal K* is obtained. The RL algorithm is shown in Table 1.

**TABLE 1:** Reinforcement-learning algorithm.

RL Algorithm	
1	Select an admissible policy *u*_*e*_ = *K*^0^ + φ;
2	For *k* = 0,1, 2⋯, given *K^k^*, collect online data, calculate δ_*XX*_, *I*_*XX*_, and *I*_*Xu*_ until the rank condition given by equation (31) is satisfied, and then solve out ${\bar{P}}^{k}$, *K^k + 1^*;
3	Improve control policy *u*_*e*_ = *K^k + 1^X*, go to step 2 until ||*K^k + 1^*−*K^k^*|| = ε;;
4	Use *u*_*e*_ = *K^k + 1^X* as the approximated optimal policy to the system.

Remark 1: To satisfy persistently exciting condition, the probing noise φ is added to the control input signal, which is necessary to guarantee nonsingular in LS solving process.

Remark 2: In Modares et al. (2016) and Li et al. (2017), RL algorithm is employed to solve the ARE with partial knowledge of system dynamics. Specifically, $\bar{A}$ is not needed in solving process, but $\bar{B}$ is still required for policy improvement. In contrast, In this study, we referred to Jiang and Jiang (2012) and employed the RL algorithm, which only uses the online information of input and system states, to solve ARE (19) neither relying on $\bar{A}$ nor $\bar{B}$. As can be seen from the definitions of $\bar{A}$ and $\bar{B}$ in (8), one can conclude that completely both human control dynamic parameters in (7) and robotic impedance parameters in (3) are not required in our method. The convergence of the RL algorithm was proofed by Theorem 7 in Jiang and Jiang (2012). Although both this study and previous study (Jiang and Jiang, 2012) employed the RL algorithm to obtain the optimal admittance parameters for improving the HRI performance with completely unknown dynamics parameters, their study does not address HRI issue for robot.

## 3 System description

**FIGURE 3:**
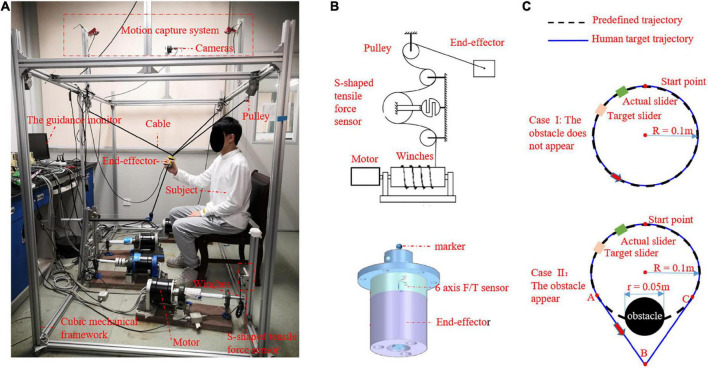
**(A)** Prototype of 3-degrees of freedom (DOFs) cable-driven rehabilitation robot (CDRR), **(B)** one cable transmission mechanism and actuator module, **(C)** the graphical guidance interface.

To perform our research on an upper-limb rehabilitation robot, a 3-DOF CDRR prototype was developed in our laboratory. As shown in Figure 3A, the CDRR constructed to demonstrate and test the proposed control strategy consisted of a cubic mechanical framework, cable transmission mechanism, actuator module, sensors, controller (MicroLabBox, dSPACE, Germany), and personal computer [intel i7-8700 CPU 3.2 G and 32 GB of random access memory (RAM), China] with ControlDesk (dSPACE, Germany) and MATLAB R2019b software. The cable transmission and actuator module consisted of four cables, pulleys, four winches, an end-effector, and four motors (DM1B-045G, Yokogawa, Japan) with servo drivers (UB1DG3, Yokogawa, Japan). The four cables were pre-stretched high stiffness and made of lightweight steel wires. One end of each cable was fastened to the end-effector and the other end was fastened to the winch. The winches were driven by the motors to control the lengths of the cables (Figure 3B). The sensors on the CDRR included S-shaped tensile/force sensors (HSTL-BLSM, Beijing Huakong Xingye Technology Company, China) mounted on the mechanical framework to measure the cable tension, a 6-axis F/T sensor (SRI-V-210105-G, Sunrise Instruments, China) attached to the end-effector to measure the human voluntary force between the CDRR and human, and a motion capture system (OptiTrack, NaturalPoint, USA) with four cameras (Flex3, NaturalPoint, USA) used to measure the position of marker placed on the end-effector. The control strategy and data acquisition and recording were implemented on the controller with sampling frequency of 1 kHz. The guidance monitor with a virtual training environment was used to design the exercise game.

## 4 Simulations studies

### 4.1 Optimization of admittance parameters through RL algorithm

Simulation studies were conducted to investigate the convergence speed and accuracy of RL algorithm. For comparison, the optimal admittance parameters were obtained using the RL algorithm and LQR algorithm (Matinfar and Hashtrudi-Zaad, 2016), respectively. According to Suzuki and Furuta (2012), we assumed that the human dynamics can be modeled as (7) with *K*_*p*_=779,*K*_*d*_=288, and *T* = 0.18 when applying the LQR method to simplify the simulations. The matrices $\bar{A}$ and $\bar{\text{B}}$ in (9) then become


$$\bar{A}=\left[\begin{array}{cccccc} 001000 & & & & & \\ 000100 & & & & & \\ 000000 & & & & & \\ 000000 & & & & & \\ 4327.8016000-5.5560 & & & & & \\ 04327.8016000-5.556 & & & & & \end{array}\right],$$

(33) $$\bar{B}={\left[\begin{array}{cccccc} 001000 & & & & & \\ 000100 & & & & & \end{array}\right]}^{T}$$

The matrices *R*_*1*_ and *R*_*2*_ in the cost function (14) were set as


(34) $$Q=d\,i\,a\,g\,\left(5000,5000,500,500,1,1\right),R_{2}=I_{2}.$$

Similar to Matinfar and Hashtrudi-Zaad (2016) and Yang et al. (2021), the optimal admittance parameters obtained directly by the LQR algorithm by considering the exact parameters of the human–robot system model (12) were


(35) $$K^{{}^{*}}=\left[\begin{array}{cc} \begin{array}{ccc} 151.459 & 0 & 58.257 \end{array} & \begin{array}{ccc} 0 & 0.810 & 0 \end{array}\\ \begin{array}{ccc} 0 & 151.459 & 0 \end{array} & \begin{array}{ccc} 58.257 & 0 & 0.810 \end{array} \end{array}\right].$$

Generally, it is nearly impossible to obtain the actual parameters of the human–robot system model (12). To avoid requiring these parameters, the RL algorithm was reformulated and fit into the optimal admittance parameters calculated online in Section “3 System description.” The initial values of the system parameters were set as


$$K_{0}=\left[\begin{array}{cc} \begin{array}{ccc} 1200 & 1400 & 1400 \end{array} & \begin{array}{ccc} 1500 & 60 & 4 \end{array}\\ \begin{array}{ccc} 1500 & 1400 & 1500 \end{array} & \begin{array}{ccc} 2000 & 70 & 10 \end{array} \end{array}\right],P_{0}=10\,{\text{I}}_{6},$$

(36) $$X_{0}={\left[\begin{array}{ccc} \begin{array}{cc} 0.1 & 0.1 \end{array} & \begin{array}{cc} 0 & 0 \end{array} & \begin{array}{cc} 0 & 0 \end{array} \end{array}\right]}^{T}.$$

To satisfy the requirement of persistent excitation, we chose a probing noise given by


(37) $$\varphi =\sum_{\omega }^{100}0.001=\left(r\,a\,n\,d-0.5\right)\,s\,i\,n\,\left(\omega \times \left(r\,a\,n\,d-0.5\right)\right)/\omega ,$$

where *rand* is a random number that varies from 0 to 1. The sampling time was selected as *T* = 0.001 and 100 samples were collected in each iteration. After 18 iterations, the optimal admittance parameters obtained by the RL algorithm were


(38) $$K=\left[\begin{array}{cc} \begin{array}{ccc} 151.462 & 0.003 & 58.257 \end{array} & \begin{array}{ccc} 0 & 0.810 & 0 \end{array}\\ \begin{array}{ccc} 0.006 & 151.464 & 0 \end{array} & \begin{array}{ccc} 58.256 & 0 & 0.810 \end{array} \end{array}\right].$$

**FIGURE 4:**
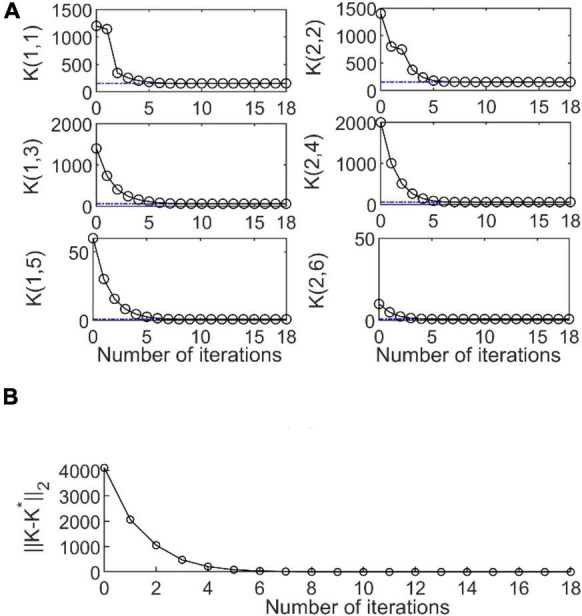
**(A)** Evolution of admittance parameters *K* for the duration of simulation, Blue lines are the optimal admittance parameters K* obtained with the linear quadratic regulator (LQR) algorithm for a specific human control dynamic model. Black lines are the results obtained by reinforcement learning (RL) method under unknown human dynamics; *K*(*i*,*j*) is the element of *K*, *i*,*andj* are index of row and column, respectively. **(B)** Convergence of ||*K*−K*|_|2_.

Figure 4A illustrates the evolution of the admittance parameters and Figure 4B show that of the error ||*K*−K*||_2_ between the RL algorithm and LQR method. After five iterations (0.5 s), the convergence errors of the optimal admittance parameters were lower than 0.01. Thus, the RL algorithm has similar accuracy as that of the LQR algorithm and acceptable convergence speed.

### 4.2 Adaptation to human dynamics

The TTOA movement task was designed which applied to across passive and active working mode for different subjects and displayed in a graphical guidance interface. As shown in Figure 3C, a predefined trajectory *P_t* (black dotted line) represented a suitable basic movement task created for patients without voluntary movement ability, which was defined as


(39) $$P_{t}=\left[0.1\,c\,o\,s\,\left(0.1\,\pi \,t+0.5\,\pi \right),0.65+0.1\,s\,i\,n\,\left(0.1\,\pi \,t+0.5\,\pi \right)\right].$$

*P_a*, which was the actual position of the end-effector, was displayed in real time with a green slider. *P_h* (blue line) was the human target path, which remained unknown to the CDRR but was displayed in real time with an orange slider in the graphical interface and can be seen by the subject. During this task, the subject was instructed to look at the green slider and orange slider in the graphical interface and control the end-effector by using her/his hand and let the green slider track the orange slider with the best performance. In order to engage and challenge patients with less severe impairments, an obstacle with a diameter of 0.05 m and center at the coordinates of O_2_(00.5500) may appear on the path of *P_t* when the orange slider reaches the point A (−0.07070.5793), and disappear when the orange slider arrives at point C (0.07070.5793) (show as Case II on the bottom row in Figure 3C). The human target path was described follows


(40) $$P_{h}=\left\{ \begin{array}{c} P_{t}\;t_{0}\leq t<t_{1}\\ A+\left(t-t_{1}\right)\,\left(B-A\right)/\left(t_{2}-t_{1}\right)\;t_{1}\leq t<t_{2}\\ B+\left(t-t_{2}\right)\,\left(C-B\right)/\left(t_{3}-t_{2}\right)\;t_{2}\leq t<t_{3}\\ P_{t}\,t_{3}\leq t<t_{4} \end{array}\right.,$$

where A (−0.07070.5793),B(00.4500),*andC*(0.07070.5793) were the joined points; *t_0* = 0 s, *t*_*1*_ = 7.5 s, *t*_*2*_ = 10.0 s, *t*_*3*_ = 12.5 s, and *t*_*4*_ = 20.0 s. When the target slider reached point A at *t*_*1*_ = 7.5 s, the subject needed to adjust its path and plan a new bypath. The arc AC moved partly into triangle ABC in *P_h* to bypass the obstacle.

**FIGURE 5:**
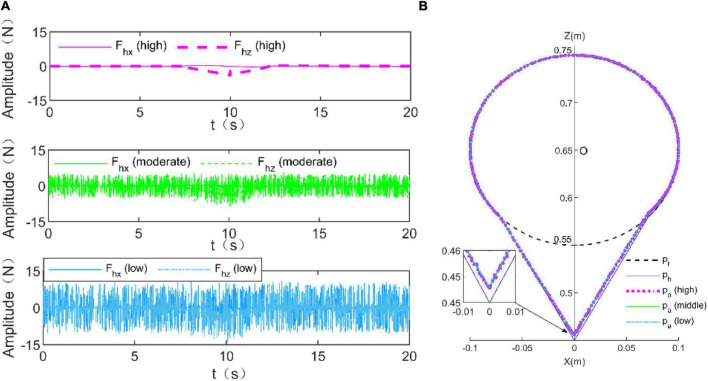
Simulation results for trajectory tracking performance under the voluntary force generated by the human dynamics models with three different levels of capabilities. **(A)** Human voluntary forces for high (violet), moderate (green), and low (blue) levels of capabilities. **(B)** Trajectory tracking performance.

In this simulation, the feasibility of the proposed RLOAC strategy was verified through simulation of the TTOA movement task. The RLOAC strategy was implemented by the method presented in Section “2 Control strategy design,” and the initial parameters were set as in the above simulation example. The parameters μ=0.5,*F*_*c*_=1.5*N*,*r*_min_=1, and *r*_max_=300 were adopted. The human dynamics model (7) was used to simulate the human voluntary force. To verify whether the proposed method can adapt itself to patients with different capabilities, three types of disturbance forces were added to the human voluntary force to simulate the movements of three types of patients with high, moderate, and low levels of capabilities (Suzuki and Furuta, 2012). Similar to Suzuki and Furuta (2012), the disturbance forces were designed as shown in Table 2. The simulation results are presented in Figure 5. Figure 5A illustrates the simulation results of the human voluntary forces exerted by patients with high, moderate, and low levels of capabilities. Figure 5B shows the trajectory tracking results under these three simulation conditions. All trajectory tracking errors were small under these three simulation conditions. Thus, the RLOAC strategy is suitable for patients with different capabilities.

**TABLE 2:** The design of three typed of disturbance force.

Levels of capabilities	Amplitude of disturbance force
High	0 N
Moderate	Random (−5 N, 5 N)
Low	Random (−10 N, 10 N)

To compare the performance of the proposed RLOAC strategy with those of other methods without online optimization of the admittance parameters, further simulation was conducted by utilizing the following traditional admittance control (TAC) to perform the aforementioned task (Culmer et al., 2010).


(41) $$P_{d}=P_{t}+F_{h}/\left(K_{T\,A\,C}+C_{T\,A\,C}\,S\right),$$

where the stiffness matric *K*_*TAC*_=*diag*(125,125) and damping matric *C*_*TAC*_=*diag*(49.4, 49.4). Both RLOAC and TAC method used the same inner loop controller. The detailed design can be referred to Yang et al. (2022) We evaluated the HRI performance in terms of the tracking accuracy and robot compliance. The absolute tracking error was used to evaluate the tracking accuracy, which was defined as follows:


(42) $$||Error\left(t\right)|{|}_{2}=||||P_{a}\left(t\right)-P_{h}\left(t\right)|{|}_{2}.$$

The energy per unit distance (EPUD) was adopted to evaluate the robot compliance (Lee et al., 2018; Zhou et al., 2021), which was defined as follows:


(43) $$E\,P\,U\,D\,\left(t\right)=\left|F_{h}\,\left(t\right)\cdot △\,d\,\left(t\right)\right|/\left|△\,d\,\left(t\right)\right|$$

where △*d* = *P*_*a*_−*P*_*t*_ is the trajectory deviation made by the subject from *P*_*t*_ to *P*_*a*_. A smaller EPUD(*t*) value indicates higher robot compliance with the human motion intentions **(Lee et al., 2018; Zhou et al., 2021).**

**FIGURE 6:**
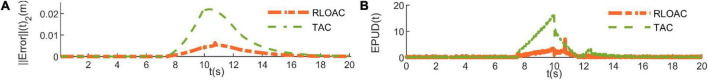
Comparison of simulation results of reinforcement-learning-based optimal admittance control (RLOAC) (orange line) and traditional admittance control (TAC) (green line). **(A)** Tracking accuracy. **(B)** Robot compliance.

The simulation results are presented in Figure 6. The tracking accuracy of the proposed RLOAC is higher than that of the TAC, especially when bypassing an unpredictable obstacle, as shown in Figure 6A. Moreover, the value of *EPUD*(*t*) needed to bypass the obstacle was notably smaller with the proposed RLOAC when compared with the TAC, as shown in Figure 6B. This comparison of the simulation results indicates that the CDRR with the proposed RLOAC achieved higher accuracy and compliance with the human motion intention.

## 5 Experimental studies

Because human control behavior and motor learning are complex and have variable characteristics, which cannot be described in the above simulations, we further investigated the validity of the proposed method through experimentation with human subjects on the 3-DOF CDRR constructed by us and illustrated in Figure 3.

### 5.1 Experimental setup

Ten healthy subjects four males and six females, age [mean (M) 28 years with standard deviation (SD) of 4.92], height (M 1.67 m with SD 4.77), and weight (M 57.05 kg with SD 7.45) with no history of neurological impairment were recruited for the experiment. All subjects provided informed consent before participating in the experiment. They were instructed to grasp the end-effector of the CDRR and perform the TTOA movement task (detailed in Section “4.2 Adaptation to human dynamics”), as shown in Figure 3C. To better show the condition of switching between passive and active working modes, the TTOA movement task was set as follows:

(1) The obstacle may appear randomly with a probability of 50%. Depending on the non-appearance or appearance of the obstacle, the task scenarios is called Case II as shown on the top row in Figure 3C or Case II on the bottom row in Figure 3C, respectively. The equation of *P*_*h*_ is different in these two cases. Specifically, *P*_*t*_ and *P*_*h*_ overlap in Case I, which are both expressed as (39). *P*_*t*_ and*P*_*h*_ only partial overlap in Case II. *P*_*t*_ is expressed as (39), while *P*_*h*_ is expressed as (40).

(2) The task of Case I and Case II were conducted periodically by performing one cycle per period.

Initially, the experimenter demonstrated the TTOA movement task to the subjects and ensured that each subject understood the task. Then, the subjects were allowed to practice 20 unrecorded cycles/period. After this preliminary experiment, to ensure a fair comparison, the same experimental protocol was conducted for two trials by each subject: once with TAC and once using the proposed RLOAC in a random order unknown to the subject. For each control strategy (TAC or RLOAC), each subject executed 10 cycles/period, including five cycles/period for Case I and five for Case II, and the data were recorded for analysis. Further, Case I and Case II appeared in a random order unknown to the subject.

### 5.2 Data analysis

We performed a quantitative evaluation of the HRI performance based on the following measures:

(1) Mean absolute tracking error (MATE), defined as


(44) $$M\,A\,T\,E=\frac{1}{s_{j}}\,\sum_{i=1}^{s_{j}}{||P_{a}\,\left(t_{i}\right)-P_{h}\,\left(t_{i}\right)||}_{2},$$

where *P*_*a*_(*t*_*i*_) and *P*_*h*_(*t*_*i*_) represented the actual position and the human target position of the end-effector at the *ith* sampling instant, respectively; and *s*_*j*_(*j* = 1,2,3) were the total number of samples for each subject during the entire experiment, the active working mode, and the passive working mode, respectively.

(2) The EPUD for each subject, defined as


(45) $$E\,P\,U\,D=\sum_{i=1}^{s_{j}}\left|F_{h}\,\left(t_{i}\right)\cdot △\,d\,\left(t_{i}\right)\right|/\sum_{i}^{s_{j}}\left|△\,d\,\left(t\right)\,\left(t_{i}\right)\right|$$

where *F*_*h*_(*t*_*i*_) and △*d*(*t*_*i*_) represented the human voluntary force and the trajectory deviation at the *ith* sampling instant, respectively.

We employed paired-samples *t*-tests with a significance level of **α** = 0.05 to test differences in MATE and EPUD of the 10 subjects between the two control strategies (TAC and RLOAC) (Losey and O’Malley, 2018). All statistical analyses were performed using SPSS 19 (SPSS, Inc., Chicago, IL, USA).

## 6 Experimental results

**FIGURE 7:**
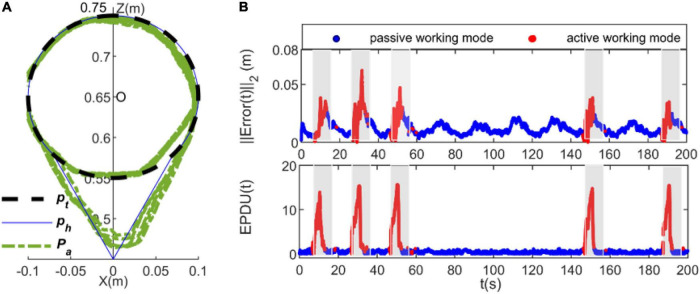
Experimental results of the proposed traditional admittance control (TAC) strategy measured from subject 2. **(A)** Tracking trajectories, **(B)** tracking error and EPUD(t). The shadows in each subplot indicate the time duration of appearance of the unpredicted obstacle and the intervention required from the participant to bypass it. The deep blue scatterplot shows the results of the passive working mode, and the red scatterplot shows the results of the active working mode.

**FIGURE 8:**
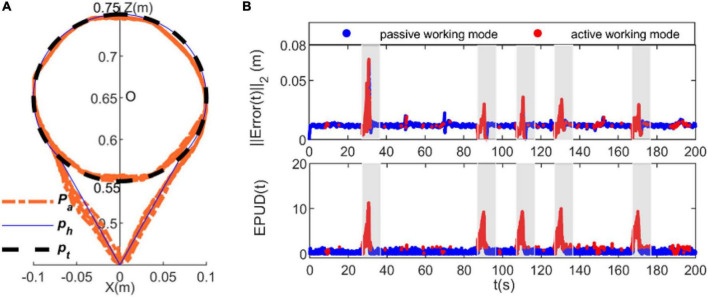
Experimental results of subject 2 when using the proposed RLOAC. **(A)** Tracking trajectories, **(B)** tracking error and EPUD(t).

**FIGURE 9:**
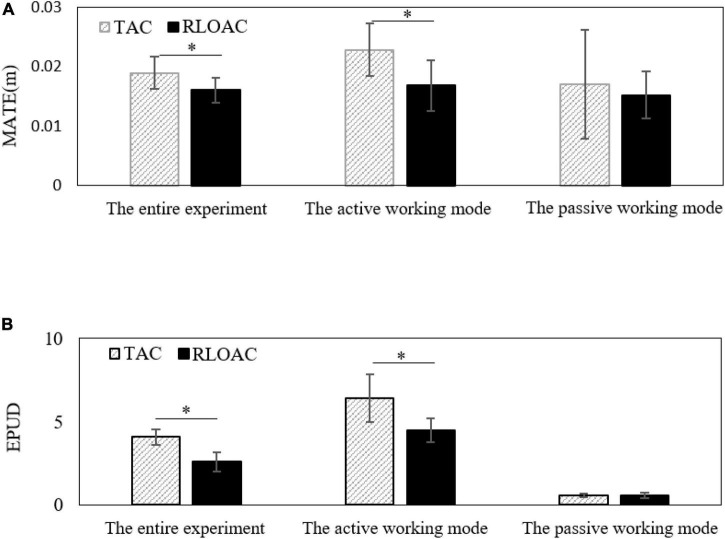
Comparison of human–robot interaction (HRI) performance in term of the mean absolute tracking error (MATE) **(A)** and energy per unit distance (EPUD) **(B)** between the proposed reinforcement-learning-based optimal admittance control (RLOAC) and traditional admittance control (TAC) in the entire experiment, the active working mode, and the passive working mode, respectively. Each plot shows the mean value for the subjects when TAC was used (grid) and when the proposed RLOAC was used (black). The error bars indicate the standard deviation, and the asterisk “*” indicates *p* < 0.05.

Our findings are presented in Figures 7–9. Figure 7 depicts the results from the subject 2 when using the TAC strategy, whereas Figure 8 depicts the results of the subject 2 when using the proposed RLOAC strategy. Specifically, Figures 7A, 8A illustrate the tracking trajectories of 10 cycles for the TAC and the proposed RLOAC strategy, respectively. The first and second rows of Figures 7B, 8B illustrate the tracking errors and the EPUD(*t*), respectively. The deep blue scatterplot shows the results for active working mode, and the red scatterplot shows the results for passive working mode. The shadows in each subplot indicate the time durations for which the obstacle appeared.

It is clear from Figure 8 that with the proposed RLOAC strategy, the CDRR can assist the subject cooperatively by complying with her/his motion intention to complete the movement task with desirable performances in terms of the accuracy and compliance. As shown in the first row of Figures 7B, 8B, the tracking error, ||*Error*(*t*)||_2_, when using the RLOAC strategy was small and acceptable, and it was mostly notably lower than that of the TAC strategy. As seen in the second row of Figures 7B, 8B, the compliance indicated by *EPUD*(t) for the RLOAC strategy was below 13 in each case, which was notably better than that of the TAC strategy. The red sections in of Figures 7B, 8B show that the active working mode time for the proposed RLOAC strategy was notably longer than that for the TAC strategy for performing the same movement task. As seen in the triangular part of the tracking trajectories, by using the RLOAC strategy, the CDRR can comply with the subjects’ voluntary actions and bypass unpredictable obstacles with greater accuracy and compliance than that possible with the TAC strategy. When using the RLOAC strategy, the tracking error and vibrations of the end-effector can be decreased through voluntary control by the subject, as it ensures better compliance and smooth switching between passive and active working mode. Even without obstacles in the path, the active working mode can be adopted to decrease the tracking error and vibrations. On the contrary, with the TAC strategy, the active working mode is rarely adopted in the absence of obstacles. Thus, the results of the representative subject show that the proposed RLOAC strategy has better accuracy and compliance, and can promote active working mode in comparison with the TAC strategy.

Figure 9 depicts the results for all 10 subjects in the form of mean ± std to statistically detect the differences between the two control strategies. As shown in Figure 9, the MATEs for the entire experiment of 10 cycles/period, the active working mode, and the passive working mode were (0.0160 ± 0.0021, 0.0168 ± 0.0043, and 0.0152 ± 0.0039) m and (0.0189 ± 0.0027, 0.0228 ± 0.0044, and 0.0170 ± 0.0091) m with the RLOAC and TAC strategies, respectively. By comparing the trials of RLOAC and TAC, there were statistically significant decreases in the MATE for the entire experiment (*p* = 0.015) and during the active working mode (*p* = 0.001). These differences were not statistically significant during passive working mode (*p* = 0.693). A similar pattern was discovered for the EPUDs. The detailed EPUDs of the RLOAC trials for the entire experiment, the active working mode, and the passive working mode were (2.5931 ± 0.5740, 4.4704 ± 0.7217, and 0.5805 ± 0.1470), whereas in the TAC trials, the corresponding EPUDs were (4.0754 ± 0.4845, 6.4994 ± 1.4368, and 0.5569 ± 0.1137). The results of the RLOAC trials were significantly smaller than those of the TAC trials for the entire experiment (*p* = 0.001) and during the active working mode (*p* = 0.006), whereas the EPUDs of these two control strategies during the passive working mode had no significant differences (*p* = 0.560). Thus, the statistical quantification analysis proved that the proposed RLOAC strategy had desirable accuracy and compliance, which were statistically notably better than those of the TAC strategy in the comparison experiment.

## 7 Discussion and conclusion

In this study, an RLOAC strategy is proposed for a CDRR that can achieve continuous mode adaptation between the passive and active working modes. Experiment with 10 subjects were conducted on a self-designed CDRR, and the results demonstrated the effectiveness of the proposed control strategy. It is demonstrated that the proposed approach can potentially be applied in CDRR.

The RLOAC strategy improved the HRI performance in terms of tracking accuracy and robot compliance. The tracking error (Modares et al., 2016; Li et al., 2017, 2018) and EPUD (Lee et al., 2018; Zhou et al., 2021) are common performance indexes of the HRI. A smaller tracking error indicates that the subjects can control the CDRR’s motion more accurately (Modares et al., 2016; Li et al., 2018), and a smaller EPUD indicates higher robot compliance (Lee et al., 2018; Zhou et al., 2021). There was decrease in the means of absolute tracking error and EPUD during exercise, because the CDRR with RLOAC can obtain the suitable admittance parameters to optimize HRI performance. Significant differences between the RLOAC and TAC strategy were found in the performance metrics during both active working mode and in the overall experiment, because the contributions to the control task between subject and robot can be adjusted as necessary for rapid adaptation to the changes in human voluntary actions and task requirements. Thus, the CDRR with RLOAC exhibited high levels of compliance with human motion intentions and self-adaptive optimization to human dynamics. The controller type did not have a statistically significant effect in the passive working mode, because the tracking error was mainly determined by the inner loop position controller in this working mode. The increase in time spent in active working mode indicates that the RLOAC strategy can promote voluntary engagement during exercise. Because the subjects participated in the control loop, and their voluntary force were utilized to perceive their motion intentions (Li et al., 2018). Continuous mode adaptation according to subjects’ voluntary force facilitated subjects driving the robot at their will made them feel in control during exercise, which may increase their motivation and confidence to use the affected limb (Proietti et al., 2016).

Comparing the RLOAC strategy with the traditional control strategies highlights its advantages. The well-recognized TAC strategy, widely applied in rehabilitation robots, was chosen as a comparison method because both the RLOAC and TAC yield the desired trajectories based on the human input forces using an admittance model to obtain robot compliance. The fixed admittance parameters were adopted in the TAC strategy, which meant that it could not adapt to the variability of human dynamics. In contrast, using reinforcement learning, the RLOAC can obtain suitable admittance parameters to optimize HRI performance. In contrast to most optimization algorithms, the RLOAC strategy can adjust admittance parameters online without the knowledge of human and robot dynamics models. Although adaptive impedance control has been applied to optimize interaction performance, as pointed out in Riener et al. (2005), Culmer et al. (2010), Proietti et al. (2016), and Zhou et al. (2021), admittance control is more stable than impedance control. Therefore, the RLOAC strategy is more suitable for rehabilitation robots due to using admittance control. Use of RL algorithm to obtain the optimal admittance parameters and optimal HRI performance by minimizing a cost function has been suggested in previous studies (Modares et al., 2016; Li et al., 2017). However, in their studies, a partial knowledge of the system dynamics was still required. In contrast, the RL algorithm in this study was improved and used to address the HRI issue considering completely unknown human and robot dynamics parameters. Moreover, continuous and real-time mode adaptation was realized by dynamically adjusted contribution weight of the cost function according to the human voluntary force.

The limitations present in this study can be given as follows. We assumed that the human voluntary force can be directly measured by a 6-axis F/T sensor. In fact, the measured force was the interaction force between the human and the end-effector, which is composed of both voluntary and involuntary components. The applicability and clinical effectiveness of the proposed control strategy was not verified in post-stroke patients.

## Data availability statement

The raw data supporting the conclusions of this article will be made available by the authors, without undue reservation.

## Ethics statement

Ethical review and approval was not required for the study on human participants in accordance with the local legislation and institutional requirements. The patients/participants provided their written informed consent to participate in this study.

## Author contributions

RY and RS contributed to the conception of the control algorithm. RY designed and performed the simulations and experiments and wrote the first draft of the manuscript. All authors contributed to the manuscript revision and approved the submitted version.
